# Efficacy and safety of Kanglaite injection for gastric cancer: A protocol for systematic review and meta-analysis

**DOI:** 10.1097/MD.0000000000021619

**Published:** 2020-08-07

**Authors:** Daorui Hou, Liangjun Yang, Jian Xiong, Lu Xiong

**Affiliations:** a Department of Traditional Chinese Medicine Oncology, The First People's Hospital of Xiangtan City, Xiangtan 411101, Hunan Province; b Department of Gastroenterology, Tongde Hospital of Zhejiang Province, Hangzhou 310012, Zhejiang Province; c Department of Oncology, Guang’anmen Hospital, Beijing, 100053, China.

**Keywords:** Kanglaite, gastric cancer, protocol, systematic review and meta-analysis

## Abstract

**Background:**

Kanglaite injection is a broad-spectrum anti-tumor drug, which is extracted from the seeds of the Chinese medicinal herb Coix lacryma-jobi, and has been widely used for the treatment of gastric cancer (GC). This study aimed to systematically investigate the efficacy and safety of Kanglaite injection for the treatment of GC.

**Methods:**

We will perform the comprehensive literature search in English and Chinese electronic database from its inception to June 2020. Two trained researchers will independently select the qualified studies for data extraction and assess the quality and risk of bias. Cochrane Risk of Bias tool will be used to assess the risk of bias of included studies. The outcomes included overall response rate, complete response rate, 3-year progression–free survival rate, 3-year overall survival rate, and different types of treatment-related adverse events. Funnel plot analysis and Egger test will be used to assess the publication bias. Finally, the quality of evidence will be assessed by the grading of recommendations assessment, development, and evaluate system . We will calculate the risk ratio as well as their 95% confidence intervals of these outcomes and pool the results using RevMan 5.4 software and STATA 16.0 software.

**Results:**

The results of our research will be published in a peer-reviewed journal.

**Conclusion:**

The conclusion of our systematic review will provide evidence to judge whether Kanglaite injection is an effective intervention for patient with GC.

**OSF registration number:**

10.17605/OSF.IO/HF679.

## 1 Introduction

Gastric cancer (GC) is the fifth most frequently diagnosed cancer and the third leading cause of cancer death, which was responsible for over 1,000,000 new cases in 2018 and an estimated 783,000 deaths.^[1,2]^ Despite recent advances in therapeutic methods including surgery combined with chemotherapy and radiotherapy, the prognosis for advanced GC patients remains very poor.^[3]^ With high incidence and mortality rate, GC causes a serious health burden globally, especially in several Western Asian and Eastern Asia countries (eg, in Mongolia, Japan, and the Republic of Korea).^[2,4]^ Thus, implementation of a multimodality treatment approach is of great significance to further improve survival.^[5]^

Traditional Chinese medicine has been used for thousands of years and is widely used as an alternative or combined treatment for cancers.^[6–9]^ Various natural products from Chinese herbal extracts exhibit strong inhibitory properties against carcinogenesis.^[10]^ As one of famous Chinese herb, Coix seed is well known for its anti-tumor and immunomodulatory effects.^[11,12]^ In recent years, Coix seed and its extracts have been used to treat many diseases such as cancer, arthritis, hypertension, asthma, and immunological disorders.^[13–15]^

In 1995, Kanglaite injection, which is extracted from the seeds of the Chinese medicinal herb *Coix lacryma-jobi*, was approved by the State Food and Drug Administration of China (Drug Approval Number: Z10970091) for the treatment of various cancers. It is also the first Chinese medicine approved by the US Food and Drug Administration, and has been used as a biphasic broad-spectrum antitumor drug in clinical trials in the United States.^[16,17]^ Millions cancer patients in more than 2000 hospitals in China have been treated with Kanglaite injection.^[18]^ It has been widely used for the treatment of Pancreatic cancer,^[19,20]^ lung cancer,^[21,22]^ breast cancer,^[23]^ colorectal cancer,^[24]^ hepatocellular carcinoma,^[25]^ and GC.^[26,27]^ Researches showed that Kanglaite injection could effectively reverse multiple-drug resistance in cancer cells and enhance the sensitivity of tumor cells to chemotherapeutic drugs.^[17,27–29]^

With the publication of numbers of trials and clinical studies on Kanglaite injection forGC, there is an urgent need for a systematic review to assess the effectiveness and safety of Kanglaite injection in treating GC. Hereby, the aim of our study is to systematically review current available randomized controlled trials (RCTs) and to objective comment the efficacy and safety of Kanglaite injection treatment of GC.

## 2 Methods and analysis

### 2.1 Study registration

This systematic review will be conducted followed the guideline of the Preferred Reporting Items for Systematic Review and Meta-Analysis Protocols recommendations.^[32]^ This work has been registered at Open Science Framework (OSF, https://osf.io/), an open source project management that helps in the design of studies. The registration DOI of this study is 10.17605/OSF.IO/HF679.

### 2.2 Eligibility criteria

#### 2.2.1 Study design

RCTs which used Kanglaite injection or a combination of Kanglaite injection and routine pharmacotherapy as treatment measures will be eligible. Nonrandomized control studies, qualitative studies, laboratory studies, and observational study will be excluded in the review. Language will be limited to English and Chinese.

#### 2.2.2 Types of participants

Trials included adult (18 years or older) participants of any ethnic origin, gender, nationality who had GC. Individuals with other malignancies and non-primary GC will be excluded.

#### 2.2.3 Types of interventions

Interventions to be reviewed are Kanglaite injection alone or combinations with other interventions to treat the GC. When Kanglaite injection used as combinations with other treatments, the control group should also receive the same combination treatments.

#### 2.2.4 Outcomes

The primary outcomes of this analysis include overall survival and progression-free survival. Overall response rate, disease control rate, and adverse events are defined as the secondary outcomes.

### 2.3 Search strategy

#### 2.3.1 Electronic searches

To identify all relevant studies, a comprehensive electronic search of the following databases including 4 English medicals database and 4 Chinese databases will be performed from their inception to June 2020: PubMed, Embase, MEDLINE, Cochrane Library Central Register of Controlled Trials, China National Knowledge Infrastructure database, Wanfang Data Knowledge Service Platform, Chinese Scientific Journals Database, Chinese Biomedical Literature Service System (SinoMed). An example of search strategy for PubMed database that combines MeSH terms and free words will be adopted. The search strategy was as follows:

#1 Search: (“Stomach Neoplasms”[Mesh]) OR ((((((((((((((((((Neoplasm, Stomach[Title/Abstract]) OR (Stomach Neoplasm[Title/Abstract])) OR (Neoplasms, Stomach[Title/Abstract])) OR (Gastric Neoplasms[Title/Abstract])) OR (Gastric Neoplasm[Title/Abstract])) OR (Neoplasm, Gastric[Title/Abstract])) OR (Neoplasms, Gastric[Title/Abstract])) OR (Cancer of Stomach[Title/Abstract])) OR (Stomach Cancers[Title/Abstract])) OR (Gastric Cancer[Title/Abstract])) OR (Cancer, Gastric[Title/Abstract])) OR (Cancers, Gastric[Title/Abstract])) OR (Gastric Cancers[Title/Abstract])) OR (Stomach Cancer[Title/Abstract])) OR (Cancer, Stomach[Title/Abstract])) OR (Cancers, Stomach[Title/Abstract])) OR (Cancer of the Stomach[Title/Abstract])) OR (Gastric Cancer, Familial Diffuse[Title/Abstract]))

#2 Search: (((Kang-lai-te[Title/Abstract]) OR (Kanglaite[Title/Abstract])) OR (KLT injection[Title/Abstract])) OR (coix seed oil[Title/Abstract])

#3 Search: (((((((((randomized controlled trial[Title/Abstract]) OR RCT[Title/Abstract]) OR random[Title/Abstract]) OR randomly[Title/Abstract]) OR random allocation[Title/Abstract]) OR allocation[Title/Abstract]) OR randomized control trial[Title/Abstract]) OR controlled clinical trial[Title/Abstract]) OR clinical trial[Title/Abstract]) OR clinical study[Title/Abstract]

#1 and #2 and #3

#### 2.3.2 Searching other resources

In addition, we will search the reference lists of studies, systematic reviews, and conference abstracts related to Kanglaite injection and GC. Ongoing trials will be retrieved from the World Health Organization International Clinical Trials Registry Platform, Current Controlled Trials, US National Institutes of Health Ongoing Trials Register, Australian New Zealand Clinical Trials Registry, and the Chinese Clinical Trial Registry. Moreover, Google scholar, Bing scholar, and Baidu scholar will be will retrieved to find out other related literature. We will also search in OpenGrey.eu. website for potential gray literature.

### 2.4 Data collection and analysis

#### 2.4.1 Selection of studies

**Figure 1:**
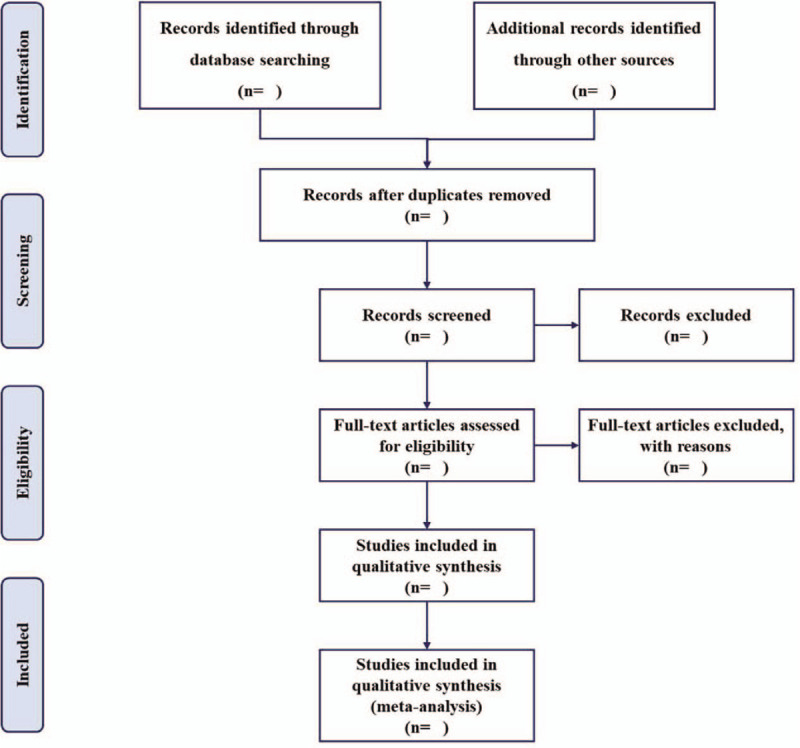
Flow chart of study selection.

The electronic citations extracted out from the above databases will be managed by Endnote X9, which is one of the most popular reference management programs among scientific community.^[30,31]^ The titles and abstracts of all searched studies will be assessed independently by 2 methodological trained reviewers (Daorui Hou and Liangjun Yang) in accordance with the established selection criteria. The full-text papers will be reviewed if necessary. Any disagreements generated between the 2 reviewers will be arbitrated through consensus with the corresponding author. Excluded studies will be listed in a table with reasons. A preferred reporting items for systematic reviews and meta-analysis flow chart (Fig. 1) will be drawn to illustrate the selection process of eligible papers.^[32]^

#### 2.4.2 Extraction and management of data

The data was extracted out by 2 independent reviewers (Daorui Hou and Jian Xiong) in accordance with the standardized sheet recommended by the Cochrane Handbook of Systematic Reviews of Interventions. The data of those qualified articles will be export to Microsoft Excel, which includes the first authors of the article, year of publication, country where the study was performed, funding, study duration, contact details of the authors, sequence generation, allocation sequence concealment, blinding, incomplete outcome data, and selective outcome reporting, participants, interventions in experimental group and control group, outcome indicators, and adverse events. Important missing data will be obtained by contacting article authors whenever possible. Data extraction will be completed independently by paired reviewers. If there is a conflict, the third reviewer (Liangjun Yang) will resolve it by organizing discussions.

#### 2.4.3 Assessment of risk of bias in included studies

Two independent reviewers (Daorui Hou and Liangjun Yang) will evaluate the risk of bias for each study using the Cochrane Collaboration's tool, an established and reliable tool for assessing the risk of bias.^[33]^ In this tool, the risk of bias of a trial is assessed through 6 items, including election bias, performance bias, detection bias, attrition bias, reporting bias, and other sources of bias. The assessment will be classified into 3 levels: “Low risk,” “High risk,” or “Unclear risk.” Disagreements between the 2 reviewers will be resolved by a third reviewer (Lu Xiong) during the evaluation of bias assessment.

#### 2.4.4 Synthesis of data

Statistical analyses will be performed using RevMan 5.4 (Cochrane, London, UK) and STATA 16.0 software. For dichotomous variables, the risk ratio will be applied to analyze. For continuous variables, a mean difference or a standard mean difference will be used for analysis. Mean difference will be used when the treatment outcome was measured by the same scale. Standard mean difference will be used when the treatment outcome was measured by different scales in different studies. The confidence intervals for both dichotomous and continuous variables will be set to 95%.

#### 2.4.5 Assessment of heterogeneity

To assess the statistical heterogeneity of evidence, the chi-squared (*X*^2^) test and the inconsistency index (*I*^2^) statistic.^[34]^ If *P* ≥ .05 and *I*^2^ ≤ 50%, it suggests that no statistical heterogeneity is observed between subgroups, and the Mantel–Haenszel fixed model will be employed for meta-analysis. If *P* < .05 and *I*^2^ > 50%, it is considered that there is great heterogeneity between the studies, and the random effect model will be used. Subgroup analysis, meta-regression, or descriptive analysis will be used for heterogeneity analysis.^[35]^ The results will be showed in tables and figures when the quantitative synthesis is not suitable.

#### 2.4.6 Sensitivity analysis

A sensitivity analysis for primary outcomes will be performed to evaluate the robustness of the review conclusions if feasible. We will exclude each study included in the analysis. Then we will re-analyze and compile the data. The difference between the re-obtained effects and the original effects will be compared.

#### 2.4.7 Grading the quality of evidence

The Grading of Recommendations Assessment, Development, and Evaluation, a widely used tool in evaluating the quality of assessment, will be applied to assess the quality level of evidence by 2 reviewers (Daorui Hou and Liangjun Yang).^[36]^ The quality of evidence will be assorted into “high,” “moderate,” “low,” and “very low” quality.^[37]^ Disagreements between the 2 reviewers will be resolved by a third reviewer (Lu Xiong).

#### 2.4.8 Publication bias

When more than 10 studies are included, a funnel plot will be used to identify the possible publication bias. Egg regression and Begger tests will be utilized to detect the funnel plot asymmetry.^[38]^*P* < .05 is considered to have publication bias.

### 2.5 Patient and public involvement

This part is not covered in this study.

### 2.6 Ethics and dissemination

This study will not need ethical approval because the data used are not linked to individual patient. The results of this review will be disseminated by being published in a peer-reviewed journal.

## 3 Discussion

GC, as a common cancer with high morbidity and mortality worldwide, requires continuous exploration for new treatment methods and concepts.^[39]^ Traditional Chinese medicine, a medicine with a history of thousands of years, has an irreplaceable role in the supplement and adjuvant treatment for GC.^[40,41]^ In recent years, Kanglaite injection has been widely used in the treatment of GC and has a significant effect on patients with GC.^[26,27]^ However, the evidence from RCTs is inconsistent. With an increasing number of clinical trials, it is urgent to systematically evaluate the efficacy of Kanglaite in the treatment of GC. In this study, we will summarize the up-to-date evidence of Kanglaite injection for the treatment of GC. This work may also provide helpful evidence to determine whether Kanglaite injection is effective and safe for patients with GC, which may benefit both clinical practice and health-related policy makers.

## Author contributions

**Conceptualization:** Lu Xiong.

**Data curation:** Daorui Hou, Liangjun Yang, Jian Xiong.

**Formal analysis:** Liangjun Yang, Lu Xiong.

**Funding acquisition:** Lu Xiong.

**Investigation:** Daorui Hou, Jian Xiong.

**Methodology:** Daorui Hou, Liangjun Yang, Lu Xiong.

**Project administration:** Lu Xiong.

**Resources:** Daorui Hou, Liangjun Yang.

**Software:** Daorui Hou, Liangjun Yang.

**Supervision:** Lu Xiong.

**Writing – original draft:** Daorui Hou, Liangjun Yang.

**Writing – review & editing:** Daorui Hou, Liangjun Yang, Lu Xiong.
